# An assessment for in vitro propagation and genetic stability of *Phoebe goalparensis* Hutchinson, an endemic valuable timber tree of North East India

**DOI:** 10.1186/s43141-023-00487-9

**Published:** 2023-03-10

**Authors:** Kalpataru Dutta Mudoi, Barbi Gogoi, Gitasree Borah, Marine Hussain, Tabassum Tasfia, Krishnakhi Borah, Himangshu Lekhak, Siddhartha Proteem Saikia

**Affiliations:** 1 Agrotechnology and Rural Development Division (ARDD), CSIR-North East Institute of Science & Technology, Jorhat, Assam 785006 India; 2 Academy of Scientific and Innovative Research (AcSIR), Ghaziabad, 201002 India

**Keywords:** *Phoebe goalparensis*, Micropropagation, Clonal fidelity, ISSR, Polymonomorphic

## Abstract

**Background:**

*Phoebe goalparensis* is an endemic forest species of North East India that belongs to Lauraceae family. *P. goalparensis* is used as timbers yielding plants for commercial importance in the local furniture markets of North East India. A rapid in vitro micropropagation protocol was established by using apical and axillary shoot tips on Murashige and Skoog medium with varied concentrations of plant growth regulators.

**Results:**

In this study, 5.0 mg/l BAP augmented medium was chosen as the best for shoot multiplication of the plant. However, IBA (2.0 mg/l) was the most responsive for root induction. Moreover, 70% of root induction was recorded during rooting experiment and 80–85% survivability was observed during the acclimatization of this species. Clonal fidelity of *P. goalparensis* was determined with ISSR marker and it was observed that in vitro raised plantlets were polymonomorphic.

**Conclusion:**

Hence, an efficient protocol with high proliferation and rooting was established for *P. Goalparensis* that could aid in massive propagation in future.

## Background

Natural resources (Forest trees) are over-exploited due to the growth of population pressure. Nowadays, a noticeable decrease is observed in the natural evergreen forest tree population of Assam. According to Assam Forest Report, 32.5 thousand hectares of forest cover in Assam has been decreased during 2009–2012 due to indiscriminate and illicit felling by the villagers and unscrupulous traders.

*Phoebe goalparensis* Hutchinson which belongs to the family Lauraceae is one of the indigenous evergreen large forest trees of Assam. It constitutes excellent light-weight and one of the most valuable timbers of Assam, known as ‘Bonsum’ in trade. It is the principal source of Bonsum timber, which is used for building structure, planking and furniture and all kinds of cabinet work, and it resembles the same properties of Teak, and hence, it is known as ‘Assam Teak’ [1]. Though it flowers profusely during April to May, almost all the ripened fruits are eaten by birds and wild animals. Hence, availability of the mature seeds is scanty and fresh seeds have 35–70% germination in the soil but germination is inhibited due to its hydrophobic nature. Moreover, seeds are recalcitrant and, therefore, cannot be stored for a long period [2]. Germination starts after about 25 days of sowing and is completed in about 4 months. Seedlings become ready for plantation after about 6 months of transplanting. Direct sowing is not found successful for this species [3]. Therefore, the above information gives a critical bottleneck in the implementation of the conservation through conventional mode for large-scale regeneration of planting material of the target species.

Under this concept, micropropagation is reported to be an effective method for the conservation of important germplasms, which can produce large-scale plantlets within a short time frame without any genetic variation [4]. Various factors like plant growth regulator, media and temperature could affect the results of micropropagation. Therefore, molecular methods such as Random Amplified Polymorphic DNA (RAPD), Restriction Fragment Length Polymorphism (RFLP), Inter Simple Sequence Repeat (ISSR) and Simple Sequence Repeat (SSR) are used for the assessment of genetic homogeneity. In this study, in vitro regeneration protocol of *P. goalparensis* for rising propagule quality from elite plant source was first attempted to establish and proliferate, and the genetic fidelity of the obtained plants was assessed. The clonal planting stocks are necessary to ensure the performance of micropropagated plantlets of *P. goalparensis.* Moreover, it is the first attempt to evaluate the in vitro culture on clonal fidelity study of *P. goalparensis*. Therefore, an approach with biotechnological tools and techniques in regeneration of quality propagates and also for ex situ conservation with sustainable exploitation of this endemic species is highly essential.

## Methods

**Fig. 1:**
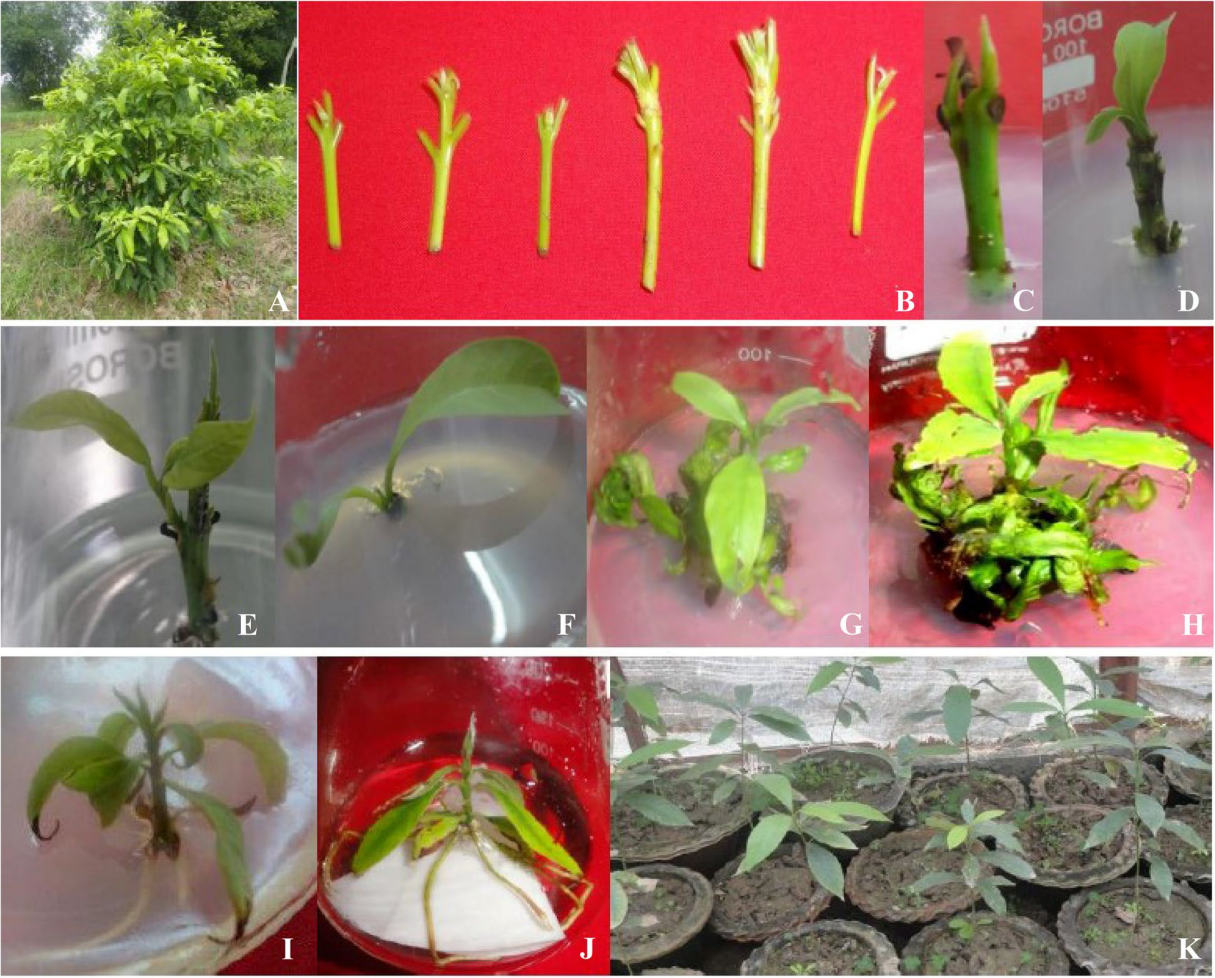
In vitro propagation of *Phoebe goalparensis*. **A** Mother plant. **B** Preparation of explants material. **C**–**F** Emergence of shoot buds. **G**, **H** Shoot multiplication. **I** Root induction. **J** Hardening. **K** Acclimatization and soil transfer

Seedlings of *P. goalparensis* Hutchinson were collected from State Forest Department of Assam and established in the field of CSIR-NEIST Experimental farm for rising of in vitro culture as a mother plant (Fig. 1A). The apical and axillary shoot buds (3–4 cm) were excised from clone number Pg7 and cleaned smoothly under tap water for removal of dust and dart (Fig. 1B). Explants’ material was treated with 5% (v/v) Tween 20 solution for 1 h and 0.3% Bavistin for 30 min followed by rigorous cleaning with running water for 4–5 times and disinfected in 70% ethanol for 1 min, 0.1% Hgcl_2_ solution (v/v) for 8 min, and then flushed 3–4 times each in germfree double-distilled water.

### Induction of in vitro shoot proliferation

Higher concentrations of TDZ causes no shoot proliferation, only lower concentration of it shows shoot proliferation [5]. Therefore, the surface-sterilized explants were cultured into BAP, Kin (0.5–5.0 mg/l) and TDZ (0.5–2.0 mg/l) enriched MS [6] medium for in vitro shoot proliferation augmented with 3% sucrose and 0.8% Agar powder from Hi Media Chemicals, India. All cultures were incubated under 16-h photoperiod at 24±2°C temperature with 35 μ mol m^−2^ s^−1^ of light power (cool white luminous light).

### Root induction

Woody Plant Medium (WPM) of Lloyd and McCown [7] was used for rooting trials. Root induction experiment was conducted with various concentrations of auxins like NAA and IBA (0.5–5mg/l). The hardening procedure was involved by few steps process, i.e. shift of rooted shoot to MS basal liquid medium for 15–20 days, which was pursued by exhibiting the rooted shoots in half strength of MS basal liquid medium for another 15 days. Roots were in liquid medium so it was washed thoroughly in running tap water to remove the traces of MS liquid medium and caps of the flasks were also removed along with periodical rinsing with antifungal solution of Bavistin fungicide (0.2% solution). After that, plantlets were kept in filtered water for total of 30 days likely 15 days in culture room and subsequently another 15 days in ambient room temperature.

### Clonal fidelity assessment of raised plantlets by ISSR

Clonal fidelity is a technique for determining the genetic stability of micropropagated plantlets before they are delivered to extensive plantations or other commercial applications. To assess the clonal fidelity, 12 in vitro raised plants were randomly selected along with its mother plant Pg7. ISSR was used to determine genetic stability and soma clonal variation of plantlets derived through micropropagation protocol. These plants were compared with the mother plant from which explants were taken. Total genomic DNA of the mother plant and in vitro raised plants was extracted from tender leaves using modified cetyltrimethyl ammonium bromide (CTAB) method [8] and quantified using Nano Drop spectrophotometer (G:BOX Syngene, UK). Initially, 25 ISSR (Genei) primers were screened and 10 ISSR primers were selected based on clear banding patterns. PCR amplification was performed in Thermal Cycler (Applied biosystem) with a total volume of 20μl reaction mixtures having 15ng/μl of DNA template, 10X PCR buffer, 2.5mM dNTPs, 5pmol primer, 1U Taq DNA polymerase (Sigma) and the final volume was adjusted with nuclease-free water. The PCR reaction programmes for ISSR were set at 94°C for 4 min followed by 35 cycles of 94°C for 30s, 90s at annealing temperature 45–50°C, according to primer’s Tm (melting temperature) then 2-min elongation at 72°C and 72°C final extension at 10 min. The amplified products were analysed in 1.5% agarose gel at 95V for 1.5h and visualized in the gel documentation system (G:BOX, Syngene, UK). Low range DNA ladder of 100bp to 1kb (Genei) was used as a standard for each marker analysis.

### Data analysis

The experiments were conducted in completely randomized design in different replication in different experiments. Experimental results were analysed statistically using the techniques of analysis of variance for single-factor experiments. The significance of the treatment means differences were tested by the procedure of Duncan’s multiple range test [9].

The scoring of ISSR was done manually in a binary format (“1” for presence and “0” for absence of bands) and distinctive bands were calculated, whereas the unclear or faint bands were not considered for further analysis. To differentiate each marker, the parameters like the total number of bands, number of polymorphic and monomorphic bands, percentage of polymorphism, polymorphic information content (PIC), resolving power (RP) and marker index (MI) were evaluated using Microsoft Excel. The value of PIC was calculated as PIC=1−∑Pi2, where pi is the frequency of *i*^th^ allele (band present) and summation extends over *n* alleles [10]. Resolving power (Rp) of each primer was estimated with Rp=∑Ib where Ib (informative fragments) = 1− [2 x (0.5−pi^2^)], where pi^2^ is the proportion of accession containing bands [11]. NTSYS (Numerical Taxonomy and Multivariate Analysis System) software v2.1 was used to generate the distance matrix and cluster analysis of the datasets [12]. A dendrogram representing the genetic association revealed by the similarity coefficient was generated using UPGMA with the Sequential agglomerative hierarchical and nested clustering method programme in NTSYS (SAHN).

## Results

### In vitro shoot regeneration

Among the various treatments, BAP (0.5–5.0 mg/l) enriched medium enhanced shoot bud proliferation in this species (Table 1). 1–3 numbers of shoot buds were induced from the cultured explants within 15–30 days of inoculation (Fig. 1C–F). 5.0 mg/l BAP augmented MS medium resulted in maximum number (5.3^a^) of shoot induction (Fig. 1G, H, Table 1). During 7–10 days of culture, shoot bud induction resulted. Lower conc. of BAP (0.5–1.0) reduced shoot bud induction. However, higher conc. of BAP resulted from an intervening callus phase at the basal portion of the explants. Effects of all three cytokinins i.e. BAP, Kin, and TDZ resulted in similar tendencies at lower and higher concentrations. But BAP manifested a notably higher effect on the number of shoot bud induction, and shoot length. MS basal medium was unresponsive towards shoot bud formation during the study.

**Table 1:** Effect of cytokinins on shoot proliferation of *Phoebe goalparensis*

Hormonal composition (mg/l)	Shoot number	Shoot length (cm)	Leaf number
Basal	0.0	0.0	0.0
BAP 0.5	2.7^cd^	1.3^fgh^	1.67^fgh^
BAP 1.0	4.0^b^	2.17^cdef^	2.33^def^
BAP 2.0	3.7^bc^	3.27^b^	3.67^bc^
BAP 5.0	5.3^a^	4.8^a^	5.3^a^
KIN 0.5	2.0^d^	1.00^h^	1.00^h^
KIN 1.0	2.3^d^	1.97^defg^	2.00^efg^
KIN 2.0	2.7^cd^	2.5^bcd^	2.67^de^
KIN 5.0	3.0^bcd^	3.07^bc^	4.3^b^
TDZ 0.5	2.0^d^	1.83^defgh^	1.67^fgh^
TDZ 1.0	2.3^d^	2.33^cde^	1.67^fgh^
TDZ 2.0	3.0^bcd^	3.03^bc^	3.67^bc^

Means are from four repeated experiments. Each treatment consisted of three replicates. Means within a row followed by the same letters are not significant at *P* = 0.05 according to DMRT

Shoot induction acquired by Kin was found to be less effective than BAP. In this experiment, the role of Kin (1.0 and 5.0 mg/l) was not significant at all. Higher conc. of BAP (5.0 mg/l) and Kin (1.0 and 5.0 mg/l) did not show any significant result towards shoot/root formation.

### Root induction

After 10 days of inoculation, root initiation was noticed in IBA (2.0 mg/l) enriched WPM medium and at this conc. the root number (6.67^a^), length of roots (2.70^a^) and root induction percentage (70%) were also higher in comparison with other conc. (Fig. 1I). But, in 4.0 mg/l IBA containing WPM medium rooting was noticed after 20 days of inoculation with retarded root growth. In case of NAA-enriched WPM medium root initiation was delayed by 25–30 days and root growth was reduced than IBA. Moreover, IAA was not at all responsive for root induction of this species. At the higher conc. of IBA (4.0–5.0mg/l), reduced percentage of rooting was observed in this species (Table 2).

**Table 2:** Effect of auxins on in vitro root induction of *Phoebe goalparensis*

Hormonal composition (mg/l)		Root number	Root length (cm)	Root induction %
NAA	IBA			
0.0	0.0	0.0	0.0	0.0
0.5		1.00^g^	0.40^e^	20
1.0		1.67^fg^	0.93^cde^	41
2.0		3.67^bc^	1.70^b^	50
3.0		3.33^bcd^	1.17^bcd^	45
4.0		2.33^ef^	1.07^bcde^	40
5.0		1.33^fg^	0.50^de^	32
	0.5	1.33^fg^	1.17^bcd^	30
	1.0	3.67^bc^	1.70^b^	45
	2.0	6.67^a^	2.70^a^	70
	3.0	4.33^b^	1.76^b^	55
	4.0	3.00^cde^	1.33^bc^	50
	5.0	2.00^fg^	1.00^bcde^	35

Means are from four repeated experiments. Each treatment consisted of three replicates. Means within a row followed by the same letters are not significant at *P* = 0.05 according to DMRT

Hardening plantlets of *P. goalparensis* were shifted to polythene sleeves containing 1:1:2 soil: sand: cow-dung mixture for acclimatization and retained in a net house for 30–40 days prior to field transfer (Fig. 1J, K).

### Clonal fidelity of tissue culture raised plantlets by ISSR

**Fig. 2:**
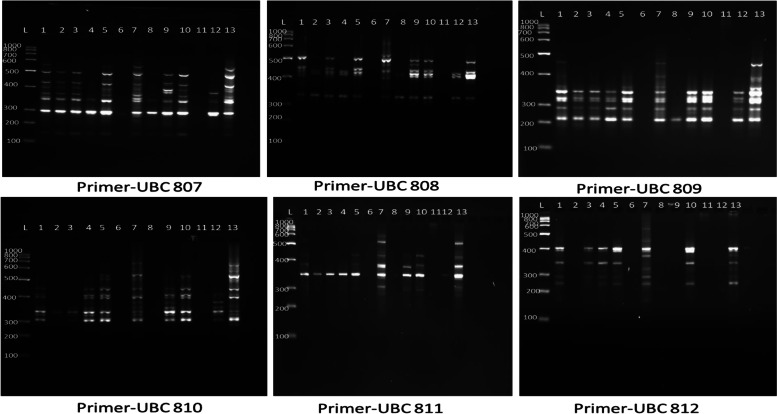
ISSR Products of *Phoebe goalparensis* amplified along with ISSR marker. **A** ISSR Primer-807. **B** ISSR Primer- 808. **C** ISSR Primer-809. **D** ISSR Primer-810. **E** ISSR Primer-811. **F** ISSR Primer-812

ISSR markers are the direct reflection of abundances and distribution of microsatellite repeat in the genome. Out of 25 screened ISSR primers, 10 anchored ISSR primers produced 73 clear bands with 13 DNA samples of micropropagated plantlets (Fig. 2). The number of amplified fragments varied from 3 to 11, with an average of 7.30 bands per primer (Table 3). Among 73 bands, 62 bands were polymorphic and the average polymorphic percentage was 85.40% across all the plantlets. Highest percentage of polymorphism (100%) was found with the primer numbers UBC812, UBC818 and UBC834, and lowest percentage (71.43%) was found in 2 primers, i.e. UBC808 and UBC809. The PIC value ranged from 0.45 (UBC807) to 0.79 (UBC812), with a mean of 0.63 per loci. The MI and Rp of the ISSR varied from 0.28 to 0.98 and 17.5 to 18.75 and the maximum MI and Rp were found in UBC810 and UBC807.

**Table 3:** Result of different genetic expressions of *Phoebe goalparensis* showing the percentage of monomorphism and Polymorphism, PIC, EMR and MI

Sl No.	Primer	Sequences	Total no. of bands	Total no. of monomorphic bands	Total No. of polymorphic bands	Percentage of polymorphism	Polymorphic information content	EMR	MI
1	807	AGAGAGAGAGAGAGAGT	8	2	6	75	0.45	4.5	2.04
2	808	AGAGAGAGAGAGAGAGC	7	2	5	71.43%	0.54	3.6	1.97
3	809	AGAGAGAGAGAGAGAGG	7	2	5	71.43%	0.54	3.6	1.95
4	810	GAGAGAGAGAGAGAGAT	11	1	10	90.90%	0.61	9.1	5.58
5	811	GAGAGAGAGAGAGAGAC	5	1	4	80%	0.75	3.2	2.43
6	812	GAGAGAGAGAGAGAGAA	6	0	6	100%	0.79	6	4.75
7	818	CACACACACACACACAG	3	0	3	100%	0.66	3	1.99
8	826	ACACACACACACACACC	8	1	7	87.5%	0.60	6.12	3.72
9	834	AGAGAGAGAGAGAGAGYT	9	0	9	100%	0.73	9	6.59
10	836	AGAGAGAGAGAGAGAGYA	9	2	7	77.78%	0.67	5.44	3.67

**Fig. 3:**
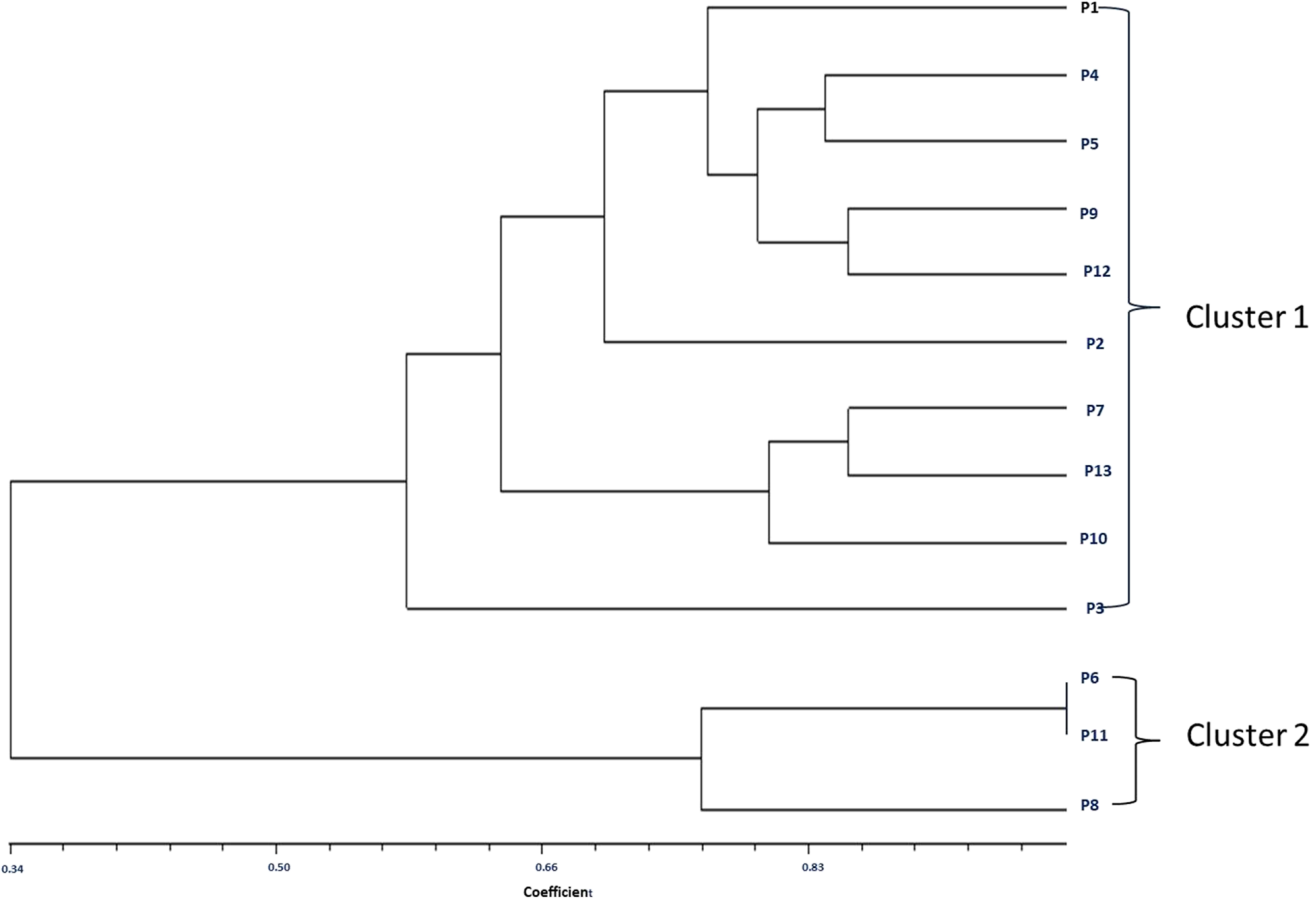
Consensus tree of *Phoebe goalparensis* accession developed on the basis of their banding patterns with ISSR Markers

The genetic similarities among the species of *P. goalparensis* were estimated according to the ISSR data. Jaccard’s coefficient showed that there were two closely related accessions, i.e. P6-P11 with the highest similarity index 0.9863014, and two distance-related accessions, i.e. P5-P11 with the lowest similarity index 0.1369863 respectively (Table 4). Cluster analysis was performed with NTSys using UPGMA method and two major clusters were generated (Fig. 3). Cluster 1 includes 10 genotypes with samples P1, P4, P5, P9, P12, P2, P7, P13, P10 and P3. Cluster 2 consists of 3 genotypes with P6, P11 and P8 samples.

**Table 4:** Similarity matrix of 12 *in vitro* raised plantlets and mother plant (Pg7) of *P. goalparensis*

	P1	P2	P3	P4	P5	P6	P7	P8	P9	P10	P11	P12	P13
P1	1.0000000												
P2	0.6438356	1.0000000											
P3	0.6164384	0.6164384	1.0000000										
P4	0.7534247	0.7260274	0.5342466	1.0000000									
P5	0.8082192	0.6712329	0.5342466	0.8356164	1.0000000								
P6	0.1780822	0.4520548	0.5616438	0.2876712	0.1506849	1.0000000							
P7	0.7671233	0.4931507	0.6027397	0.6027397	0.7123288	0.2191781	1.0000000						
P8	0.4109589	0.6575342	0.6301370	0.4931507	0.3561644	0.7671233	0.2876712	1.0000000					
P9	0.7808219	0.7260274	0.5342466	0.7808219	0.8082192	0.2328767	0.6849315	0.4657534	1.0000000				
P10	0.7397260	0.5205479	0.6575342	0.6849315	0.7397260	0.3013699	0.8082192	0.3698630	0.7671233	1.0000000			
P11	0.1643836	0.4383562	0.5479452	0.2739726	0.1369863	0.9863014	0.2054795	0.7534247	0.2191781	0.2876712	1.0000000		
P12	0.7123288	0.7397260	0.5205479	0.8219178	0.7671233	0.3287671	0.5890411	0.5068493	0.8493151	0.6986301	0.3150685	1.0000000	
P13	0.6986301	0.4520548	0.6164384	0.5342466	0.6986301	0.2328767	0.8493151	0.3013699	0.5616438	0.7945205	0.2191781	0.5479452	1.0000000

## Discussion

Micropropagation provides a feasible substitute to seed propagation as it entitles rapid propagation of elite stock cultivars in a fairly short duration of time. For raising of quality plant material, genetic consistency of micropropagated plants is a prerequisite factor. It is well known that adenine uses its ontogenesis effects on growth and organ formation of plant tissues by determining nucleic acid metabolism [13]. Efficacy of BAP towards shoot multiplication was investigated in various plant species such as *Eucalyptus* [14], *Psidium guava* [15], *Syzygiumcumini* [16], *Bambusanutans* [17], *Bambusabalcooa* [18], *Chlorophytumborivilianum* [19], *Calotropisprocera* [20], *Artemisia arborescens* [21], *Boerhaaviadiffusa* [22], *Andrographisalata* [23] and in cucurbits also [24, 25]. In our study, among the three cytokinins BAP, Kin and TDZ, BAP was the most superior, followed by Kin and TDZ. This finding was in accordance with *Curcuma caesia*, which initiated BAP was more suitable for shoot proliferation than Kin and TDZ [26, 27]. Similarly, BAP was mentioned as the most potent cytokinin for shoot induction and multiplication. The efficacy of BAP over other types of cytokinin in shoot multiplication might be due to the ability of the plant to metabolize BAP more readily as nucleotides, and ribosides stability is present naturally in BAP [28]. In contrast to our results, the regeneration potentiality of Kin is at least a fold and a half more than the percentage of regeneration using the other three types of cytokinin. The varied physiological response of using Kin than the other types of cytokinin was reported earlier. In *Cucumissativus*, Kin gives less than twofold of increasing number of shoots/explant than the other types of cytokinin with 1.0 mg/1 concentration. When 2.0 mg/1 concentration of TDZ was used, the regenerates using Kin, with a concentration of 1.0 mg/l, is more by 1.7 fold, which differed from our result [29].

It is prevalent that auxins play a pivotal role in the assurance of rooting capacity, which is crucial for vegetative propagation. The inclusion of exogenous auxin resolves the issue of difficult to root woody plant species and has been described to be imperative for root formation in various species like Oak [30], Chestnut [31] and Poplar [32]. However, the refreshing effect of IBA on rooting was earlier described in *Excoecariaagallocha* [33], *Meliaazedarach* [34], *Bambusa vulgaris* [35] and *Eucalyptus globulus* [36]. Contrary to our study, efficacy of NAA towards promoting root induction was well explained earlier [37–39]. Moreover, prior studies reported that high concentration of auxin inhibits shoot growth along with root elongation and stimulates cell differentiation [40].

In contrast, genetic instability occurs in the in vitro regenerated plants (somaclonal variation) due to use of hyper-optimum potency of growth regulators and continuous sub culturing. ISSR marker was used in order to detect genetic stability of in vitro raised 12 plantlets of *P. goalparensis* with the mother plant (Pg7). Allelic composition of 12 tissue culture raised plantlets and its mother plant were polymonomorphic. This result suggested somaclonal variation in *P. goalparensis*, which interfered with the integrity of the regenerated clonal plantlets. Somaclonal variation amid in vitro propagation may occur from pre-existing variations, the type of explant accustomed, the concentration and type of growth regulator in the medium, the number and duration of subcultures, effect of stress, genotype and the method of propagation espoused. The concentration of synthetic plant growth regulators in the medium is also associated with somaclonal variation [41]. Moreover, the preparation of many explants from only one donor plant augments the chances of variation in cultures [42].

The results of the ISSR marker system in the present study revealed the genetic variability among the in vitro raised plantlets of *P. goalparensis.* PCR contours of the 12 micropropagated plantlets for 11 ISSR primers exhibit polymorphism among themselves and with the mother plant. Bhatia et al. estimated clonal variation among the *Gerbera* plants by using ISSR marker regenerated from leaf explants [43]. The exact cause of variation might be due to explant source or due to mode of plant regeneration [44], media composition, or culture conditions and sub- or supra-optimal levels of plant phytohormones [45]. Still, somaclonal variation was reported for a numerous of in vitro derived plant species, such as *Populus deltoides* [46], *Populus tremuloides* [47], *Prunus persica* [48], *Actinidia deliciosa* [49], grapevine cv. Crimson Seedless [50], *Solanum tuberosum* [51], etc.

Unlike our findings, genetic stability was reported in several cases, namely, *Picea mariana* plants regenerated from somatic embryogenesis [52], micropropagated shoots of *Pinus thunbergii* Parl [44]., adventitious shoots of *Pinus taeda* [53], axillary bud proliferation of chestnut rootstock hybrids [54] and tissue culture-derived plantlets of Almond [55] and sugarcane [56].

## Conclusion

A high shoot multiplication rate for rooting and reproducible protocol for micropropagation of *P. goalparensis* was established. This study indicates that 5mg/l BAP augmented medium was the best for shoot multiplication and ISSR analysis accessed the genetic stability of in vitro raised 12 plantlets and suggests that the genetic variability among the in vitro raised plantlets of *P. goalparensis* could be used in the future for large-scale propagation.

## Data Availability

The data are available within the manuscript and also accessible from the corresponding author upon request.
